# Supplementary material for: Impact of Ready-Meal Consumption during Pregnancy on Birth Outcomes: The Japan Environment and Children’s Study
Source: Nutrients. 2022 Feb 20;14(4):895. doi: 10.3390/nu14040895 (PMC8877490; doi:10.3390/nu14040895)
Supplement: Supplementary file 1 [file nutrients-14-00895-s001.zip › nutrients-1600910-supplementary.pdf]

Table S1. Association of perinatal abnormality and intake frequency of ready-cooked foods (Crude)

		Stillbirth (≥ 12 wk gestation)[a] n = 842		Pre-term birth (< 37 wk gestation)[b] n = 4547		Small for gestational age infant n = 6599		Low birth weight (< 2500 g) n = 7601	
		q-value	ORs (95% CI)	q-value	ORs (95% CI)	q-value	ORs (95% CI)	q-value	ORs (95% CI)
**Ready-made meals (cooked with microwave heating in general)**	**< once a week**		1.000		1.000		1.000		1.000
	**1-2 times per week**	0.000	3.217 (2.371-4.364)	0.000	1.155 (1.08-1.234)	0.071	0.940 (0.888-0.995)	0.461	1.029 (0.976-1.085)
	**≥ 3-7 times per week**	0.000	4.336 (2.633-7.138)	0.754	1.030 (0.916-1.158)	0.811	0.986 (0.9-1.079)	0.527	1.040 (0.953-1.136)
**Frozen meals (cooked with microwave heating in general)**	**< once a week**		1.000		1.000		1.000		1.000
	**1-2 times per week**	0.000	3.404 (2.633-4.4)	0.017	1.108 (1.029-1.193)	0.754	1.015 (0.956-1.078)	0.144	1.054 (0.994-1.117)
	**≥ 3-7 times per week**	0.000	2.950 (1.934-4.498)	0.195	1.090 (0.98-1.213)	0.626	1.032 (0.947-1.125)	0.525	1.039 (0.956-1.129)
**Retort pouch foods (heated with boiling water in general)**	**< once a week**		1.000		1.000		1.000		1.000
	**1-2 times per week**	0.000	2.266 (1.801-2.851)	0.004	1.131 (1.05-1.219)	0.471	1.032 (0.97-1.098)	0.002	1.109 (1.047-1.176)
	**≥ 3-7 times per week**	0.634	0.708 (0.264-1.896)	0.461	0.878 (0.688-1.121)	0.754	1.042 (0.868-1.251)	0.172	1.154 (0.975-1.364)
**Convenience foods in plastic container (heated with boiling water in general)**	**< once a week**		1.000		1.000		1.000		1.000
	**1-2 times per week**	0.000	2.369 (1.783-3.147)	0.005	1.132 (1.049-1.223)	0.340	1.044 (0.977-1.115)	0.024	1.083 (1.019-1.151)
	**≥ 3-7 times per week**	0.598	0.742 (0.348-1.583)	0.754	0.953 (0.769-1.18)	0.804	0.969 (0.811-1.157)	0.842	1.022 (0.868-1.203)
**Canned foods (without heating in general)**	**< once a week**		1.000		1.000		1.000		1.000
	**1-2 times per week**	0.129	1.516 (0.97-2.368)	0.004	1.212 (1.079-1.362)	0.862	0.989 (0.893-1.095)	0.090	1.100 (1.002-1.207)
	**≥ 3-7 times per week**	NA	NA NA	0.451	0.714 (0.391-1.305)	0.804	0.925 (0.593-1.443)	0.936	0.979 (0.65-1.475)
**Beverage (can or plastic bottle)**	**< 7 times per week**		1.000		1.000		1.000		1.000
	**7-13 times per week**	0.000	5.312 (4.042-6.981)	0.001	1.140 (1.062-1.224)	0.753	0.984 (0.928-1.043)	0.037	1.069 (1.012-1.13)
	**≥ 14 times per week**	0.000	4.176 (2.671-6.527)	0.000	1.360 (1.227-1.507)	0.027	1.122 (1.026-1.227)	0.000	1.187 (1.092-1.291)
**Beverage (coffee bean or tea leaf)**	**< 7 times per week**		1.000		1.000		1.000		1.000
	**7-13 times per week**	0.000	4.811 (3.79-6.107)	0.020	1.104 (1.026-1.188)	0.005	1.101 (1.037-1.169)	0.021	1.078 (1.019-1.141)
	**≥ 14 times per week**	0.008	1.799 (1.236-2.62)	0.754	0.981 (0.899-1.07)	0.000	1.155 (1.079-1.236)	0.471	1.034 (0.968-1.103)

The odds ratio was calculated with the data complemented by the multiple imputation method. [a] Early miscarriage (< 12 weeks' gestation) and artificial abortion were excluded from analyses. [b] Miscarriage and artificial abortion were excluded from analyses.
